# Other PHAC publications

**DOI:** 10.24095/hpcdp.44.9.10

**Published:** 2024-09

Researchers from the Public Health Agency of Canada also contribute to work published in other journals and books. Look for the following articles published in 2024:

Djiadeu P, Begum H, Archibald C, Ekmekjian T, Busa G, Dansoh J, VanNguyen P, Merckx J, Fleurant A. Risk of transmission of HIV to infants during breast/chest feeding when mothers/birthing parents living with HIV are on antiretroviral therapy: a protocol for a rapid review. BMJ Open. 2024;14(5). https://doi.org/10.1136/bmjopen-2024-084436

Essar MY, Wahdati S, O’Sullivan B, Nemat A, Blanchet K. Cycles of disasters in Afghanistan: the urgent call for global solidarity. PLOS Global Public Health. 2024;4(1). https://doi.org/10.1371/journal.pgph.0002751

Frehlich L, Turin TC, Doyle-Baker P, Lang JJ, McCormack GR. The relationship between neighbourhood built characteristics, physical activity, and health-related fitness in urban dwelling Canadian adults: a mediation analysis. Prev Med. 2024;185. https://doi.org/10.1016/j.ypmed.2024.108037

Lange S, Llamosas-Falcn L, Kim KV, Lasserre AM, Orpana H, Bagge CL, et al. A dose-response meta-analysis on the relationship between average amount of alcohol consumed and death by suicide. Drug Alcohol Depend. 2024;260. https://doi.org/10.1016/j.drugalcdep.2024.111348

Toigo S, Betancourt MT, Prince SA, Colley RC, Roberts KC. Sociodemographic differences in recreational screen time before and during the COVID-19 pandemic in Canada. Health Rep. 2024;35(5):3-15. https://doi.org/10.25318/82-003-x202400500001-eng

